# The importance of regional availability of health care for old age survival - Findings from German reunification

**DOI:** 10.1186/s12963-015-0060-2

**Published:** 2015-09-29

**Authors:** Tobias C. Vogt, James W. Vaupel

**Affiliations:** Max Planck Institute for Demographic Research Konrad-Zuse-Str. 1, 18057 Rostock, Germany

**Keywords:** Life expectancy, Circulatory mortality, Health Care Quality, Germany, Natural experiment

## Abstract

**Background:**

This article investigates the importance of regional health care availability for old age survival. Using German reunification as a natural experiment, we show that spatial variation in health care in East Germany considerably influenced the convergence of East German life expectancy toward West German levels.

**Method:**

We apply cause-deleted life tables and continuous mortality decomposition for the years 1982–2007 to show how reductions in circulatory mortality among the elderly affected the East German catch-up in life expectancy.

**Results:**

Improvements in remaining life expectancy at older ages were first seen in towns with university hospitals, where state-of-the-art services became available first.

**Conclusion:**

Our results suggest that the modernization of the health care system had a substantial effect on old-age life expectancy and helped to significantly reduce circulatory diseases as the main cause of death in East Germany.

## Introduction

There is no definitive answer to the question of whether increased spending on health care results in better health outcomes. Although there is a general association between rising life expectancy and increasing health care spending over time, it is less clear whether rising medical investments are really the trigger for improving population health. The quality and efficiency of health care delivery are more important than the level of spending per se for improvements in health and survival [1–3]. Nevertheless, the high costs incurred by the delivery of state-of-the-art medicine seems to pay off in reducing mortality from cancer and circulatory diseases [4, 5]. Here we contribute to this discussion. We investigate whether rising investments in the modernization of the health care system in East Germany contributed to improving life expectancy levels.

The reunification of Germany provides an opportunity to assess the impact of political and socioeconomic changes on human mortality. German reunification may be viewed as a large-scale natural experiment: two populations with a shared culture and history experienced very different political and socioeconomic conditions for more than four decades. The unforeseen fall of the Berlin Wall in November 1989 suddenly terminated this separation, and in less than a year political union was achieved. Within a few years, East Germans experienced a deep transformation of their society and adopted the social, economic, and political framework of West Germany.

## The case of East Germany

**Fig. 1:**
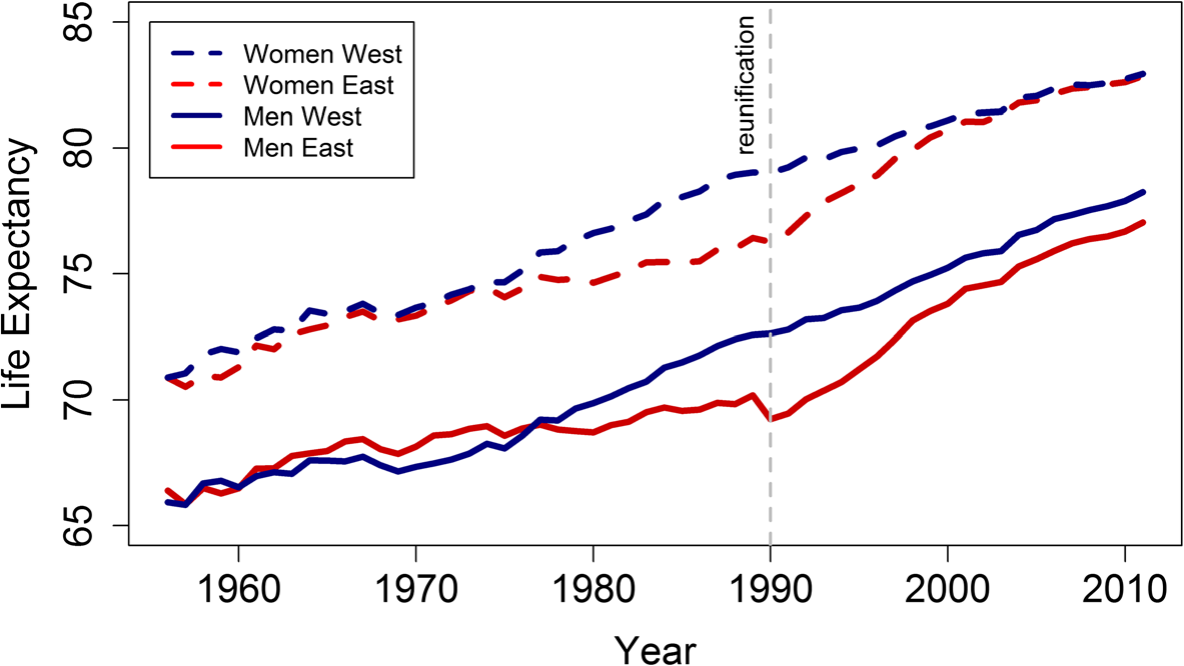
Life expectancy at birth in East and West Germany (Source: Human Mortality Database. www.mortality.org)

In the two decades following the reunification of Germany, East Germans experienced remarkable increases in life expectancy. Between 1990 and 2009, average life expectancy rose 6.3 years among women and 7.4 years among men. Over the same period, average life expectancy among West Germans increased substantially but more modestly by 3.5 years for women and 5.1 years for men. Consequently, the gap in life expectancy between the East and West narrowed from 2.7 years for females and 3.4 years for males in 1990 to 0.04 and 1.1 years in 2009 [6] [Fig. 1].

Mortality differentials between Eastern and Western European countries increased during the 1970s and 1980s, mainly because of the inability of Eastern European countries to reduce circulatory diseases among the elderly [7–9]. After the fall of the Iron Curtain, Central European countries were particularly successful in reducing mortality at older ages [10–14]. Apart from changing lifestyle factors involving smoking and nutrition, the availability of modern western health care has been proposed as the dominant driver of the catch-up process [15–21].

**Fig. 2:**
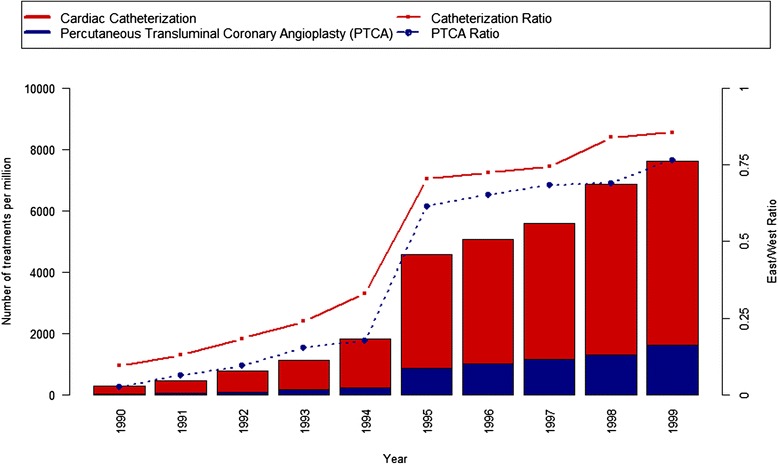
East-West ratio and number of cardiac catheterization and percutaneous transluminal coronary angioplasty treatments per one million persons (Source: German Heart Reports 1990-1999)

This analysis aims to provide greater insight into the role of the change in health care provision as a pivotal determinant in rising life expectancy in East Germany. To show the impact of the availability of modern health care, we focus on the catch-up process in university towns compared to the rural areas and smaller towns of East Germany. We assume that modern medical technologies reached rural areas later than in cities. State-owned university hospitals, located in a few major cities, benefited first from public investments in medical infrastructure and modern health care services, whereas it took some time for a network of specialized private physicians to be established in the East [22, 23]. Consequently, we argue that if modern medicine played a role in the convergence of life expectancy, then mortality would decline first in areas where modern medicine became available first. Unfortunately, data on the regional level of medical treatment do not exist for East Germany for the years around reunification. To illustrate the lower medical standard in the East we therefore have to rely on other relevant indicators. Figure 2 represents the availability of two key treatments for the prevention of cardiovascular mortality. In contrast to other indicators like hospital beds, these indicators show how many patients received a certain treatment and how different age groups were affected.

The graph shows the West-East ratio of cardiac catheterization facilities and the number of percutaneous transluminal coronary angioplasty treatments per one million inhabitants between 1990 and 1999. It demonstrates that there was a comparative undersupply of cardiovascular treatments in East Germany, mainly due to the lack of adequate modern facilities. This changed quickly after reunification. In 1990, 13 percutaneous transluminal coronary angioplasty treatments per million inhabitants were carried out in East Germany while in West Germany 527 per million received this treatment. This means that the supply was 40 times higher in West Germany. By 1999, the last year for which we have information on the difference in East-West supply, 1,612 per million inhabitants received percutaneous transluminal coronary angioplasties in the East and 2,106 in the West. In 1990, there were five specialized hospitals for these cardiac interventions of which all were state owned and four among them were university hospitals [24]. Seven years later, these treatments were available in more than 30 medical facilities across East Germany and only eight of those were in cities with university hospitals.

**Fig. 3:**
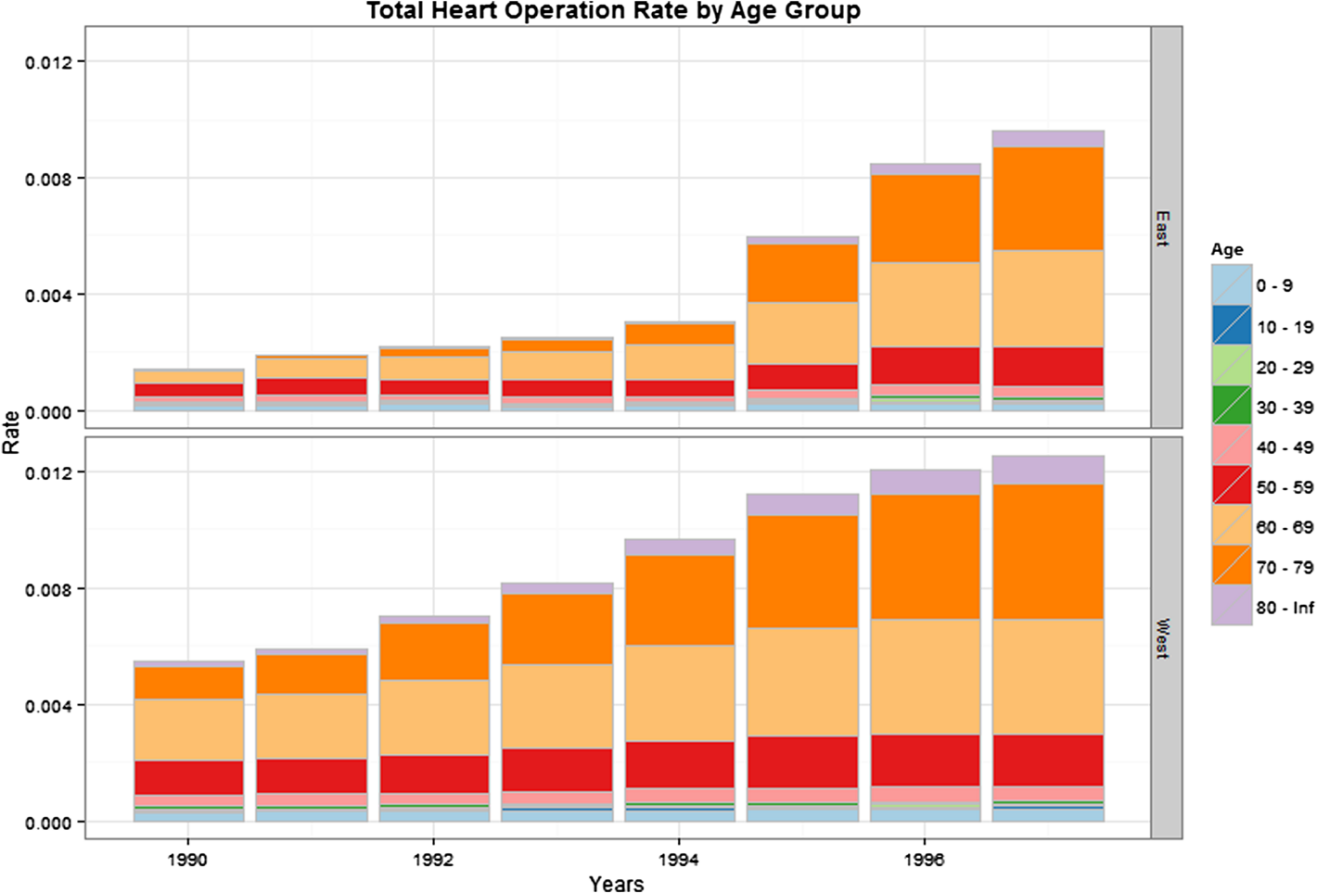
Number of operations per 1,000 persons received by age group (Source: German Heart Reports 1990-1999)

At the same time, more and more older East Germans received cardiac interventions (Fig. 3). In 1990, only 0.46 heart operations per 1,000 inhabitants were performed on East Germans over the age of 60 while West Germans above this age received 3.37 heart operations per 1,000 inhabitants. Only seven years later, older Germans in the East had 7.41 heart operations per 1,000 inhabitants compared to 9.55 in the West.

## Data

In order to test our hypotheses, we use the official cause-of-death statistics provided by the statistical offices of the five East German states for the years 1982–2007. The statistics comprise register data for population numbers and causes of death for both sexes in East Germany at the district level. To compare the development of mortality in East vs. West Germany, we used death and population counts from the Human Mortality Database and circulatory death counts from the German Federal Statistical Office. The data are organized into 5-year age groups up to the age of 85 + .

The 25 years of observation enable us to analyze the changes that occurred as a consequence of German reunification. To permit regional comparisons of mortality improvements, the dataset is split into three regions: first, all cities in East Germany with a university hospital^1^; second, all districts in East Germany without university hospitals; and third, West Germany as a whole. Our definition of circulatory disease mortality is based on the WHO International Classification of Diseases (ICD). Until 1997, the analysis included all circulatory diseases covered by the WHO ICD 9^th^ Revision, Chapter 349-459. From 1998 onwards, mortality from failures of the circulatory system was estimated by including all diseases within Chapter I00–I99 in the 10^th^ Revision of the WHO ICD. By including these diseases, we cover important causes of death that are referred to as amenable diseases or ‘unnecessary untimely deaths’ that could have been successfully treated by medical interventions [20, 25].

## Methods

Based on these data, we were able to account for mortality improvements before and after reunification for all districts in East Germany and for both sexes. This enabled us to discern the immediate impact of the political transformation. Single-decrement and cause-deleted life tables were calculated for each year to estimate remaining life expectancy and progress in reducing mortality from cardiovascular diseases for each age group in every district. The cause-deleted life tables account for life expectancy in the absence of circulatory mortality; i.e. they show the years that would have been gained if circulatory mortality had been avoided. We also applied age and cause-of-death decomposition to determine the extent to which single age groups contributed to the life expectancy differentials between the eastern regions and West Germany as a whole [26]. The decomposition reveals the specific impact of each age group and of circulatory disease mortality on the mortality convergence after reunification in each region.

## Results

**Fig. 4:**
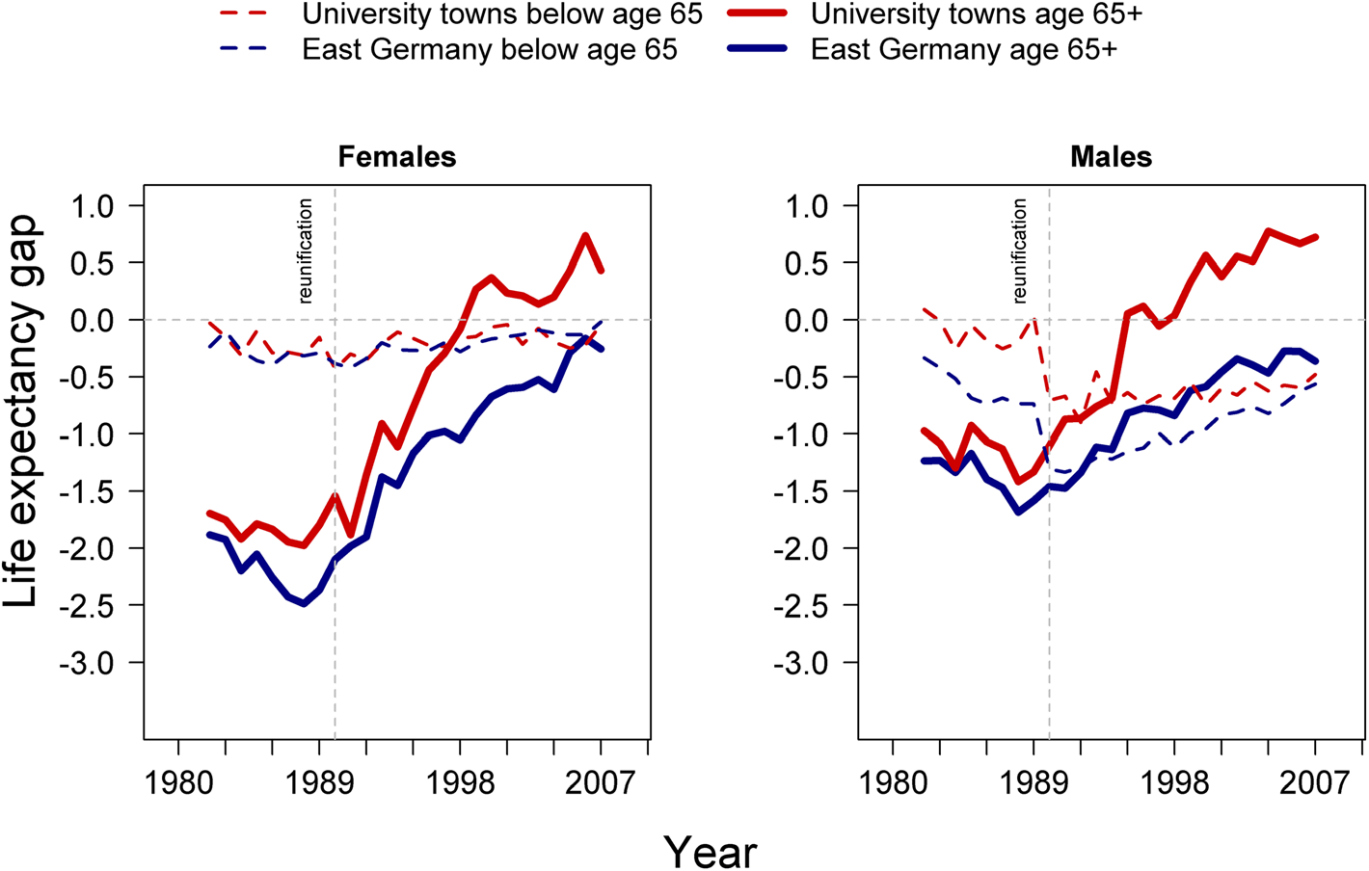
Remaining and partial life expectancy in East Germany and East German towns with university hospitals compared to West German levels for the age groups above age 65 and below age 65

Both men and women in East German university towns caught up with West German life expectancy faster than men and women in the rest of East Germany (Fig. 4). The results differ by age. While East Germans above age 65 reached the West German mean life expectancy quickly, the gap in partial life expectancy for those under age 65 remained or even increased.

Between 1982 and 2007, remaining life expectancy among older females over age 65 in East German university towns rose from 15.2 years to 20.9 years compared with an increase from 15.0 years to 20.2 years among older women in the rest of East Germany. For eastern German men over age 65, remaining life expectancy increased from 12.3 years to 17.7 years among those who were living in university towns and from 12.0 years to 16.6 years among those who were living in the rest of East Germany. Most of these increases in life expectancy occurred after the fall of the Berlin Wall in 1989. During the 1980s, East German men and women over age 65 gained around 0.5 years of additional life expectancy. In the years following the fall of the Iron Curtain, men living outside university cities gained 3.9 additional years of life, and women gained 4.4 additional years. Men over age 65 living in university towns gained 4.7 additional years of life expectancy and women gained 4.5 additional years.

The gains in life expectancy occurred at different paces. Before 1990, remaining life expectancy at age 65 stagnated all over East Germany and fell further behind the constantly rising West German level. In the year of reunification, the mortality differentials between university towns and the other districts of East Germany were smaller than the gap to West Germany. In 1990, the intra-East German gap in remaining life expectancy above age 65 was 0.3 years for men and 0.6 years for women. Yet the gap with West Germany for males was 1.1 years in university towns and 1.4 outside university towns. For East German females the gaps were even bigger. In 1990, women above age 65 lagged 1.5 years behind in university towns and 2.1 outside university towns. These gaps started to disappear during the 1990s. East German women above age 65 who were living in university towns had caught up to the West German level by 1998 and have since had a higher remaining life expectancy. Women in the same age group in the rest of East Germany also followed this trend, but did not reach the western average remaining life expectancy until years later. In 2007, their remaining life expectancy was only 0.02 years lower than the West German average. This general pattern also holds true for men. East German men above age 65 who were living in university towns reached the West German level earlier than men in the rest of East Germany. Since 1995, the remaining life expectancy of East German men in university towns has exceeded the West German average. By 2007, this advantage had risen to 0.7 additional years of life expectancy. In contrast, elderly East German men living outside university towns still have a 0.4 years lower life expectancy than men of the same age in West Germany.

Trends in life expectancy for younger East Germans are different. While older females and males in the East have caught up with their counterparts in the West, younger East Germans have not. The partial life expectancy for younger females between age 0 and 65 was only around 0.3 years below the average Western life expectancy in the 1980s. After reunification women across East Germany converged slowly to the West German level independent of their place of residence. While females and older age groups caught up to the West, men below the age of 65 fell further behind during the transformation years. Before reunification, male partial life expectancy for men living outside university towns started to lag behind. Simultaneously, men in university towns kept pace with the West German increases in partial life expectancy. More striking is the sudden widening of the difference in East-West German life expectancy between 1989 and 1991. In these two years, men below the age of 65 living in university towns and other East German districts fell behind by 0.7 years. During the following years, life expectancy for those men living outside university towns started to recover but remained a half year below the West German average.

**Fig. 5:**
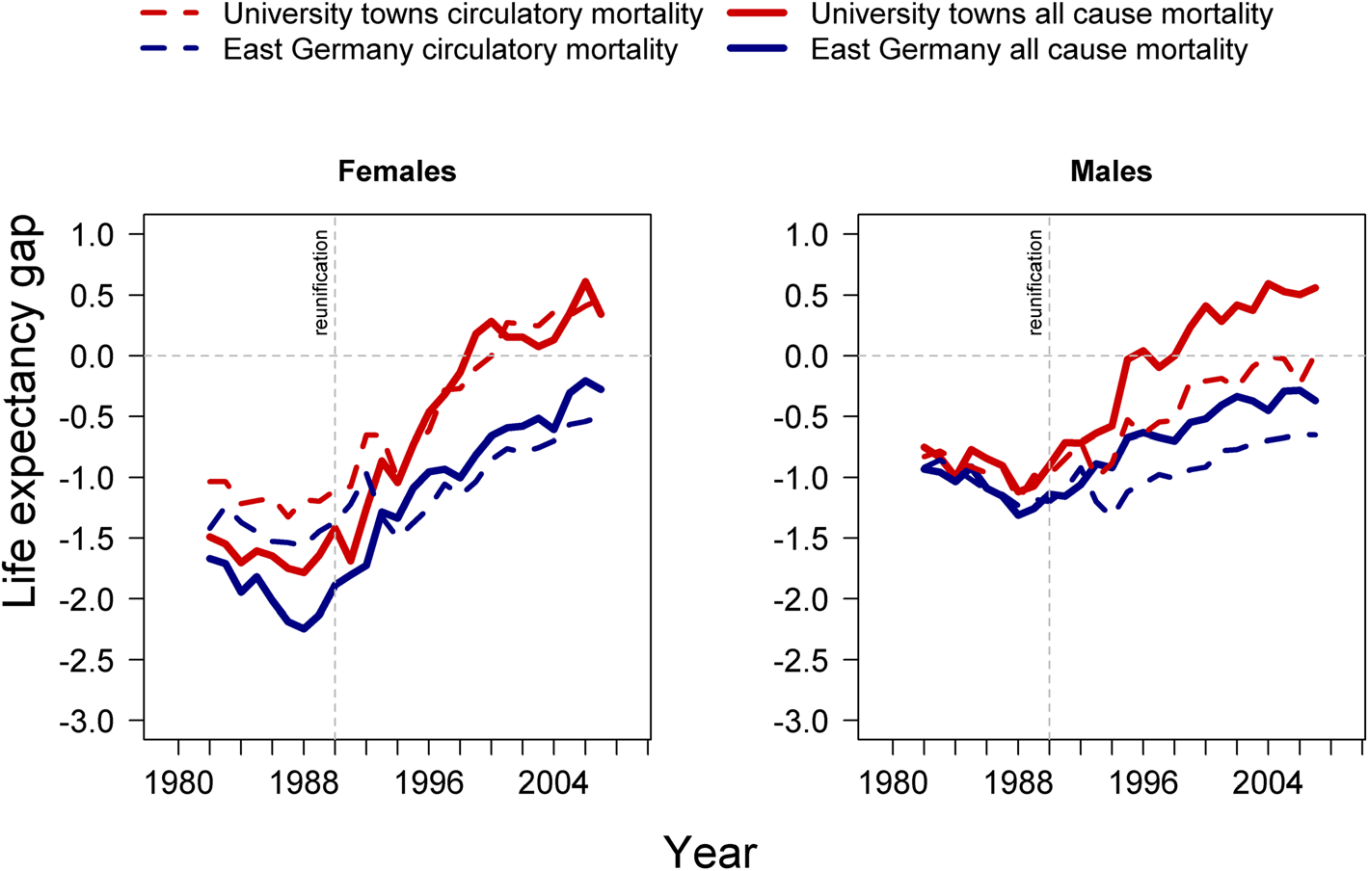
Gap in years lost to circulatory mortality. East German females and males above age 65 in university towns and outside university towns in comparison to West Germany

The rapid convergence of life expectancy after reunification is mainly due to circulatory mortality improvements among the elderly. Figure 5 shows the impact of circulatory mortality and its contribution to the East-West German life expectancy gap for the age group over 65 years.

Circulatory mortality before reunification accounted for 75 to 85 % of the female and 90 to 100 % of the male life expectancy differential between East and West Germany. During the 1980s, the female East-West mortality differential for causes of death other than circulatory diseases remained relatively stable at around -0.5 years, but the circulatory mortality differential increased from around -1.5 years to around -2.3 years. After reunification, circulatory mortality in university towns declined markedly and has been lower for females than for the West German average. East German women living outside university towns caught up at a slower pace but had almost closed the gap with the West German level by the end of 2007. They still lose 0.5 more years of life expectancy to circulatory diseases than the West German average.

The reduction of circulatory mortality is not the exclusive cause but it is an important driver of the more rapid mortality convergence to the West German level among men in the East. Before reunification, circulatory mortality accounted for the entire life expectancy gap between East and West Germany; the increasing gap resulted from the widening circulatory mortality differential. Between 1982 and 1990, this differential increased from -0.5 years to -1.5 years. Yet circulatory mortality among elderly men in university towns declined rapidly after reunification, reaching the West German level in the early 2000s. Mortality from causes other than circulatory diseases was already lower than the average West German level from the mid-1990s. Men living in areas without immediate access to modern health care were less successful in catching up to the West German level. Despite considerable progress in reducing mortality, they were still lagging behind by half a year in 2007.

## Discussion

Our results for East German university towns suggest that the availability of modern, western-style health care facilities played a pivotal role in rising life expectancy in East Germany after reunification. We were able to confirm the hypothesis that mortality first declined in areas where state-of-the-art medical services were provided first, comparing towns with university hospitals to the rest of East Germany. East Germans living in university towns experienced an accelerated decline in mortality and therefore a more rapid convergence towards the West German mean life expectancy.

The increases in life expectancy were mainly driven by progress at older ages in reducing circulatory mortality. Women and men living in university towns pulled ahead of the rest of East Germany. Older males and females outside university towns also made gains in catching up to West German life expectancy, but a gap in life expectancy due to higher circulatory mortality remains.

Remarkably, there were no marked differences between university towns and other East German regions in pre-reunification life expectancy levels. The higher density of better educated individuals in university towns did not result in higher life expectancies. The catch-up for the elderly only started after reunification. In contrast to the life expectancy convergence for the elderly, we found that East German men under the age of 65 suddenly fell back around reunification and have hardly recovered. Younger men in university towns suffer the same mortality disadvantage as men in other East German regions.

The general lag in mortality improvements in East Germany before reunification was probably due to the inadequate delivery of medical services to the elderly. From the 1970s onwards, the elderly suffered from the inability of the eastern health system to keep pace with western medical innovations and with the improved but capital-intensive treatment of chronic diseases [27]. Additionally, shortages of nursing homes, of regular medical check-ups, and of rehabilitation services were widespread. At the time of reunification in 1990, it is estimated that medical technology lagged behind by 15 to 20 years [28]. Medical infrastructure improved first in state-owned university hospitals, as modern medical technologies first became available there. Consequently, diseases became treatable, and the elderly, who had previously received inadequate treatment, benefited greatly from new therapies. This assumption is well in line with findings that show how investments in medical interventions can help to improve old age health and survival [29].

The comparable trends in partial life expectancy during the 1980s seem to reflect the setting of sociopolitical priorities in East Germany in favor of younger ages and a corresponding neglect of the elderly. While older East Germans benefited from the changes post-reunification, younger men may have suffered from the transformation. Our results show that the persistent gap in male life expectancy between eastern and western Germany is caused by men below the age of 65. Twenty-five years after German reunification their life expectancy lags even further behind than during the 1980s. This does not necessarily mean that they did not benefit from the availability of modern health care but the effect may have been outweighed by other factors. They may have suffered from the economic transformation, especially from high rates of unemployment and the outmigration of more educated individuals to West Germany. Surprisingly, younger females were less affected by these changes. Even though they were hit comparably hard by the massive lay-offs and outmigration, their partial life expectancy remained stable and kept pace with the West German average. One important reason might be that younger East German men exhibited very unfavorable health behaviors. They smoked more tobacco and drank more alcohol than West German men [30, 31]. This is not the case for older East Germans and particularly not for younger women, who smoked less than women in the West [32, 33]. In contrast, the rapid improvements in remaining life expectancy for the older age groups suggest that changes in health behaviors may not have been the dominant driver of reductions in cardiovascular mortality. Changes in smoking habits or alcohol consumption seem to have gradual, long-term effects on population-level health. Moreover, East and West German cohorts that reached age 65 during the 1990s experienced similar smoking and alcohol consumption patterns [33, 34]. Despite its relative importance, health care is certainly not the only reason why life expectancy improved. The catch-up to the West German level started right after reunification when the majority of East Germans did not have access to state-of-the-art facilities. Income levels and living standard rose after the social, economic and currency union between East and West Germany on July 1, 1990. In particular, East German pensioners benefitted from a substantial income increase with the introduction of the West German public pension system; this may have helped them to improve their standards of living and health [35].

To disentangle the relative importance of these changes and to answer the question why younger men still lag behind is beyond the scope of our study and cannot be addressed in detail here. A more important shortcoming is that we had to rely on an indirect approach to analyze the importance of health care. Individual level data on health care usage before reunification do not exist for East Germany and are confidential under West German legislation. In 1991, more than 100 public health insurance companies were operating in East Germany, and more than 1,000 in West Germany, which makes accessing these data challenging [36]. Additionally, the relationship between the amount of health care spending and the direct effect on health and mortality remains controversial [37, 38]. Increasing health care expenditure does not necessarily relate to improved health; it can also reflect economic inefficiencies or simply cost increases. This may have also been the case in East Germany after reunification. The association, however, between improvements in medical infrastructure and the decline of mortality seems to indicate a direct link between health care and improved survival.

## Conclusion

Our analysis used the impact of German reunification to show that improvements in health care quality contributed to rising life expectancy levels among East German men and women. Remaining life expectancy for the age groups above 65 first converged to the West German level in areas where modern health care was available first, with reductions in circulatory diseases as the main driver. Our findings from a natural experiment imply that mortality can be improved rapidly, even at older ages, if modern medical treatments become available. This is in line with results from studies showing that regional mortality levels are lower in areas with an efficient and good-quality health care supply.

## Footnotes

1 The cities of Rostock, Greifswald, Halle, Magdeburg, East Berlin, Leipzig, Dresden, and Jena had a university hospital before and after reunification.
